# Supplementary material for: Triple Synchronous Primary Malignant Tumors of the Liver, Kidney, and Lung in a Male Patient: Case Report and Systematic Review
Source: Diagnostics (Basel). 2025 Dec 12;15(24):3172. doi: 10.3390/diagnostics15243172 (PMC12731358; doi:10.3390/diagnostics15243172)
Supplement: Supplementary file 1 [file diagnostics-15-03172-s001.zip › Supplementary_Material_S3_CARE_Checklist.pdf]

CARE case report guidelines

# CARE Checklist (2013) of information to include when writing a case report

(cc) BY-NC-ND

| Topic | Item | Checklist item description | Reported on Page |
|---|---|---|---|
| **Title** | 1 | The words “case report” should be in the title along with the area of focus | 1 |
| **Key Words** | 2 | 2 to 5 key words that identify areas covered in this case report | 1 |
| **Abstract** | 3a | Introduction—What is unique about this case? What does it add to the medical literature? | 2 |
| | 3b | The main symptoms of the patient and the important clinical findings | 5, 6, 7 |
| | 3c | The main diagnoses, therapeutics interventions, and outcomes | 6, 7, 8, 9 |
| | 3d | Conclusion—What are the main “take-away” lessons from this case? | 10 |
| **Introduction** | 4 | One or two paragraphs summarizing why this case is unique with references | 9 |
| **Patient Information** | 5a | Demographic information and other patient specific information | 5, 6 |
| | 5b | Main concerns and symptoms of the patient | 5 |
| | 5c | Medical, family, and psychosocial history including relevant genetic information (also see timeline). | 5, 6 |
| | 5d | Relevant past interventions and their outcomes | 5 |
| **Clinical Findings** | 6 | Describe the relevant physical examination (PE) and other significant clinical findings | NO |
| **Timeline** | 7 | Important information from the patient’s history organized as a timeline | 5 |
| **Diagnostic Assessment** | 8a | Diagnostic methods (such as PE, laboratory testing, imaging, surveys) | 6, 7 |
| | 8b | Diagnostic challenges (such as access, financial, or cultural) | 6 |
| | 8c | Diagnostic reasoning including other diagnoses considered | 6 |
| | 8d | Prognostic characteristics (such as staging in oncology) where applicable | 6 |
| **Therapeutic Intervention** | 9a | Types of intervention (such as pharmacologic, surgical, preventive, self-care) | 8 |
| | 9b | Administration of intervention (such as dosage, strength, duration) | 8 |
| | 9c | Changes in intervention (with rationale) | NO |
| **Follow-up and Outcomes** | 10a | Clinician and patient-assessed outcomes (when appropriate) | 8 |
| | 10b | Important follow-up diagnostic and other test results | 8 |
| | 10c | Intervention adherence and tolerability (How was this assessed?) | NO |
| | 10d | Adverse and unanticipated events | NO |
| **Discussion** | 11a | Discussion of the strengths and limitations in your approach to this case | NO |

| | | | |
|---|---|---|---|
| | **11b** | Discussion of the relevant medical literature | 3, 4, 5, 9, 10 |
| | **11c** | The rationale for conclusions (including assessment of possible causes) | 5, 6, 7 |
| | **11d** | The primary "take-away" lessons of this case report | 9, 10 |
| **Patient Perspective** | **12** | When appropriate the patient should share their perspective on the treatments they received | NO |
| **Informed Consent** | **13** | Did the patient give informed consent? Please provide if requested | **Yes** |
