# Supplementary material for: Triple Synchronous Primary Malignant Tumors of the Liver, Kidney, and Lung in a Male Patient: Case Report and Systematic Review
Source: Diagnostics (Basel). 2025 Dec 12;15(24):3172. doi: 10.3390/diagnostics15243172 (PMC12731358; doi:10.3390/diagnostics15243172)
Supplement: Supplementary file 1 [file diagnostics-15-03172-s001.zip › Supplementary_Table_S1_Data_Extraction.pdf]

Supplementary Table S1. Published cases of synchronous triple primary malignancies (n = 83). Data extracted from PubMed, Embase, and Scopus (search period: 24 Aug 2024 – 11 Sep 2025). Only histologically confirmed, synchronous malignant tumors within two months were included.

Author & Year	Patient Details (Age, Risk factors, Symptoms)	Investigations	Diagnoses	Structures affected	Treatment	Reported Survival Outcome	Ref
**Digestive system**							
A. Allan et al., 1993	70 M<br>Epigastric pain<br>Absolute constipation<br>Epigastric tenderness	X-ray = colonic obstruction<br>Laparatomy	Moderately differentiated adenocarcinomas (1 at the mid-transverse colon = obstructive, 2 at the splenic flexure)	Colon	Colonic resection with ileo-sigmoid anastomosis	Not specified	[1]
	66 F<br>Central abdominal pain<br>Absolute constipation	X-ray = colonic obstruction	Moderately differentiated adenocarcinomas (upper rectum, lower sigmoid colon, transverse colon)	Colon<br>Rectum	Colonic resection with ileorectal anastomosis	Not specified	
K. W. Schmid et al., 1988	79 F<br>acute abdominal pain<br>right upper quadrant tenderness	US = gallstones, gallbladder wall thickening<br>Laparotomy = suspicious gallbladder mass, enlarged appendix, transverse colon mass	Moderately differentiated adenocarcinoma of the colon<br>Early adenocarcinoma of the appendix<br>Highly differentiated adenocarcinoma of the gallbladder	Colon<br>Appendix<br>Gallbladde r	Resection of the transverse colon<br>Appendectomy<br>Cholecystectomy	Not specified	[2]
F. M. Sauri et al., 2021	61 M<br>Vague abdominal complaints	CT = descending colon and esophagus wall thickening,<br>Colonoscopy = descending colon mass	Adenocarcinoma of the stomach<br>Squamous cell carcinoma of the esophagus<br>Adenocarcinoma of the descending colon	Esophagus<br>Stomach<br>Colon	Endoscopic mucosal resection<br>Esophagectomy<br>Adjuvant chemotherapy (5-FU + cisplatin)<br>Laparoscopic left hemicolectomy	Alive, 1 Year	[3]
Chien-Chih Yeh et al., 2013	79 M<br>Abdominal pain<br>Hematochezia<br>Poor appetite<br>Weight loss<br>RU quadrant tenderness	CT = bowel wall thickening, mesentery inflammation in the hepatic flexure of the colon and cecum<br>Colonoscopy = rectal tumor<br>Laparoscopy = a colon mass at the hepatic flexure and a cecal mass	Moderately differentiated rectal adenocarcinoma<br>Adenocarcinoma of the colon	Colon<br>Rectum	Low anterior resection<br>Extended right hemicolectomy	Not specified	[4]
W. Jiang et al., 2023	63 F<br>Upper abdominal pain<br>Acid regurgitation<br>Anemia	Gastroscopy = gastric lesion in the antrum<br>CT = ascending colon and rectal lesion<br>Colonoscopy = rectal and colonic cancer	Poorly differentiated gastric adenocarcinoma<br>Adenocarcinoma of the ascending colon<br>Adenocarcinoma of th rectum	Stomach<br>Colon<br>Rectum	Distal gastrectomy<br>Right hemicolectomy<br>Anterior resection of the rectum<br>Adjuvant chemotherapy – capecitabine and oxaliplatin	Alive, 1 year	[5]
B. M. Boland, 2011	77 F<br>Lower abdominal pain<br>Episodes of diarrhoea<br>Family history of gastric cancer<br>Freely mobile mass in the right iliac fossa	US = right iliac fossa mass<br>CT + colonoscopy = left colonic tumour<br>Laparotomy = 3 tumours of the appendix, a Meckel`s diverticulum and the descending colon	Colonic Adenocarcinoma<br>Carcinoid of a Meckel`s diverticulum<br>Mucinous adenoma of the appendix	Appendix<br>Colon<br>Small intestine (Meckel`s div.)	Subtotal colectomy<br>Diverticulectomy	Alive, 5 years	[6]
De Rosa M. et al., 2019	75 F<br>Hematochezia<br>Abdominal pain	CT = thickening of the distal descending colon and the distal sigmoid colon<br>PET = hypermetabolic activity in the colon at the hepatic flexure	Colonic carcinoma	Colon	Total colectomy	Not specified	[7]
J. Cheng et al., 2015	51 F<br>Hematochezia<br>Abdominal pain	CT = mass in ascending and descending colon<br>Colonoscopy = sigmoid colon lesion	Moderately differentiated adenocarcinomas of the ascending and descending colon<br>Wel differentiated adenocarcinoma of the sigmoid colon	Colon	Total colectomy with ileo-rectal anastomosis	Not specified	[8]

A. Petroianu et al., 2018	43 F<br>Dysphagia<br>Epigastric pain	Endoscopy = 3 masses in the upper, middle and lower esophagus	Squamous cell carcinoma of the esophagus	Esophagus	ChT (cisplatin + fluoropyrimidine)<br>25 cycles of radiotherapy<br>Gastrostomy	Died after 11 months- undernutrition and bilateral pneumonia	[9]
P. Wang et al., 2009	61 M<br>Solid mass in the right inferior gum<br>Three-year exposure to high concentrations of benzene	CT = esophageal neoplasm, liver mass	Basaloid squamous cell carcinoma of the esophagus<br>Hepatocellular carcinoma<br>Squamous cell carcinoma of the gum	Liver<br>Gum<br>Esophagus	Surgical resection without ChT	Died after 5 months- massive hemorrhage of gastrointestinal tract induced by thrombocytopen ia	[10]
Xiaoli Zhan et al., 2021	70 M<br>choking while eating	CT= tumor in the middle of esophagus and right sided colon<br>Endoscopy= Esophageal carcinoma and gastric cancer	Advanced esophageal squamous cell carcinoma<br>Adenocarcinoma of gastric horn<br>Advanced moderately differentiated adenocarcinoma of the colon	Esophagus<br>Stomach<br>Colon	Jejunostomy postop radiotherapy<br>Resection of right-sided colonic cancer<br>Lysis of abdominal adhesions	Not specified	[11]
Abdullah Mohhame d Aloraini et al., 2018	71 M<br>Constipation and per rectal bleeding with a palpable mass<br>Diabetes mellitus	Colonoscopy= friable mass 3-9 cm from anal verge<br>MRI=enlarged lymph nodes	Rectal adenocarcinoma<br>Pancreatic tail ductal adenocarcinoma<br>Gastrointestinal stromal tumor of the stomach	Pancreas<br>Stomach<br>Rectum	Pancreatectomy<br>Splenectomy<br>Resection with colorectal anastomosis diverting loop ileostomy<br>Chemoradiation	Not specified	[12]
V. H. Chong et al., 2010	80 M<br>Weight loss<br>Leg oedema<br>Chronic hepatitis B<br>Gastrectomy for gastric adenocarcinoma 13 years prior	Colonoscopy= diverticulae in right and left colon<br>CT= cirrothic liver with splenomegaly	Hepatocellular carcinoma<br>Gastric collision tumor with adenocarcinoma<br>Large B cell lymphoma	Stomach	No specific therapy for any of the malignancies was offered	Died, 1 month after	[13]
Masahide Fukaya et al., 2013	69 M<br>Jaundice	Endoscopic retrograde cholangiopancreatography<br>Abdominal CT	Cancer of the ampulla of Vater<br>Moderately differentiated esophageal squamous cell carcinoma<br>Well-differentiated gastric adenocarcinoma	Esophagus<br>Stomach<br>Ampulla of Vater	Esophagectomy with mediastinal and cervical lymph node dissection<br>Gastrotomy<br>Pancreatoduodenectomy	Died after 1 year	[14]
Masaya Tamura et al., 2003	70 M	Endoscopy= IIa+IIc type tumor- stomach<br>Abdominal U/S= polypoid lesion of the gallbladder<br>Colonoscopy= polypoid tumor in sigmoid colon	Well-differentiated adenocarcinoma in the stomach<br>Poorly-differentiated papillary adenocarcinoma of the gallbladder<br>Adenocarcinoma of the sigmoid colon	Stomach<br>Gallbladde r<br>Sigmoid colon	Distal partial gastrectomy (D1)<br>CholecystectomyEndosco pic mucosal resection	Not specified	[15]
Shen Gouliang et al., 2013	33 M<br>Upper abdominal pain<br>Poor appetite<br>Weight loss<br>Cronic virus hepatitis B for 10 years	CT= 10x8 cm mass in the left lobe of liver with portal vein cancerous embolus<br>Colonoscopy= a mass in the right colon	Highly-differentiated adenocarcinoma of right colon<br>Partial myxoadenocarcinoma<br>Carcinoid of appendix<br>Hepatocellular carcinoma of left liver	Liver<br>Right colon<br>Appendix	Right colectomy<br>Transarterial chemoembolization (TACE) twice	Died after 8 months- liver failure	[16]
Koushiro Ohtsubo et al., 2013	77 M<br>Diabetes mellitus	U/S= mass on the pancreatic head 25mm diameter<br>Gastrointestinal endoscopy= gastric tumor<br>CT=cecal tumor	Adenocarcinoma of the pancreas stage IIA<br>Moderately differentiated adenocarcinomas of the stomach and cecum	Pancreas<br>Stomach<br>Cecum	Chemotherapy (100mg/day S-1)<br>Pancreatic enzyme replacement therapy	Died after 18 months- bleeding from gastric cancer	[17]
Koichi Sato et al., 2003	74 M<br>Epigastralgia<br>icterus	CT=dilatation of the common bile duct with a low density mass on the left side<br>Dilatated cystic duct, intrahepatic bile duct and gallbladder<br>ERP=marked dilatation of the main pancreatic duct	Poorly differentiated tubular adenocarcinoma of the common bile duct<br>Well differentiated tubular adenocarcinoma of the gallbladder<br>Mucinous cystadenocarcinoma of the pancreas	Common bile duct<br>Gallbladde r<br>Pancreas	pancreatoduodenectomy	Died after 4 months- recurrence of the cancer	[18]
B D K Leong et al., 2008	65 M<br>Epigastric pain, loss of appetite and weight<br>Melaenic stool	Colonoscopy= tumours at transverse colon and cecum<br>Caecal and transverse colon lesions identified during laparotomy	Poorly differentiated adenocarcinoma for lesions at caecum and transverse colon	Caecum<br>Transverse colon<br>Jejunum	Total colectomy with ileorectal anastomosis and segemental jejunal resection with primary anastomosis	Alive, 2 years	[19]

		20cm ulcerative constrictive lesion at jejunum	Moderately differentiated adenicarcinoma of the jejunum		Chemotherapy (fluorouracil+ folinic acid)		
Chen JH et al., 2001	72 M<br>Poor appetite<br>Epigastric pain that radiated to his back	GI endoscopy= gastric ulcer 1.5cm at the prepyloric area<br>U/S=tumor in the gallbladder<br>CT=tumor in the poaterior body of the pancreas	Gastric adenocarcinoma<br>Gallbladder adenocarcinoma<br>Stromal cell tumor of the stomach	Stomach<br>Gallbladder	Subtotal gastric gastrectomy with Billroth-II gastrojejunostomy cholecystectomy	Not specified	[20]
**Respiratory**							
Kazuki Omori et al., 2021	72 M<br>Abnormal chest roentgenogram	CT= 2.2cm nodule in S3 with suggested pleural invasion<br>Bronhoscopy=1.1am elevated lesion at B6 and 0.3sm mucosal abnormality at B1/2	Squamous cell carcinoma in all three lesions	Lung	Left S3 segmentectomy<br>Left S6 and right B1/2 radiotherapy including endobronchial brachytherapy (EBBT)	Died after 2 years and 6 months	[21]
Kashif M et al., 2017	63 M<br>Chest pain<br>Dry cough<br>Weight loss	Bronchoscopy= endobronchial lesion<br>PET=6cm mass in right upper lobe	Adenocarcinoma in the right upper lobe<br>Poorly differentiated carcinoma favoring squamous cell cancer<br>Mixed small and large cell neuroendocrine lung cancer	Lung	Cisplatin and etoposide based chemotherapy	Not specified	[22]
Hyun Jung Yoon et al., 2014	72 M<br>Abnormal chest radiograph<br>Ex smoker	Radiographs= 4cm peripheral opacity in the right lower lobe<br>CT= 4.2cm solid mass in anterior-basal segment 3.8cm lesion at the posterior basal segment	Squamous cell carcinoma with moderate differentiation<br>Invasive mucinous adenocarcinoma<br>Invasive nonmucinous adenocarcinoma	Lung	Right lower lobectomy<br>Mediastinal lymph node dissection<br>Wedge resection of the right upper lobe	Not specified	[23]
Tsutomu Koyama et al., 2021	64 M<br>Ex smoker<br>Chest discomfort<br>History of combined pulmonary fibrosis and emphysema	CT=3 abnormal nodules in the right lower lung lobe+ bilateral interstitial changes<br>PET=significant uptake in each nodule	Keratinizing squamous cell carcinoma<br>Solid predominant adenocarcinoma<br>Small cell lung cancer	Lung	CT=3 abnormal nodules in the right lower lung lobe+ bilateral interstitial changes<br>PET=significant uptake in each nodule	Alive, 1 year	[24]
Luis Gorospe et al., 2023	72 M<br>Active smoker<br>Progressive dyspnea and cough	Radiograph= nodular opacity in the left lung<br>CT=3 suspicious lesions in the superior segment of the left lower lobe<br>EBUS and bronchial brushing=no malignant cells	Low-grade malignant neuroendocrine tumor<br>Invasive acinar-predominant adenocarcinoma<br>Invasive acinar-predominant adenocarcinoma	Lung	Left lower lobectomy	Not specified	[25]
Eun Kyoung Kim et al., 2017	65 M<br>Foreign body sensation<br>Voice change	Laryngoscope examination= protruding irregular-shaped mass on the epiglotis<br>CT and MRI= supraglotic mass+lung mass in the upper lobe<br>CT=pulmonary mass in the right lower lobe	Squamous cell carcinoma (locally advanced laryngeal cancer)<br>Small cell carcinoma<br>Squamous cell carcinoma	Larynx<br>Lung	Chemoradiation therapy+ weekly cisplatin	Not specified	[26]
Riki Okita et al., 2009	71 F<br>Smoker	Radiography= nodular shadow in the left lung<br>HRCT=3 nodules in the left upper lobe	Well-differentiated Squamous cell carcinoma<br>Poorly differentiated Squamous cell carcinoma	Lung	Left upper lobectomy	Alive, 3 years	[27]
Yoshio Tokuchi et al., 2000	66 F<br>Smoker	CT=abnormal shadows in the chest<br>Bronchoscopy= 5mm tumor in the right upper lobe	Advanced large cell carcinoma<br>Two early squamous cell carcinomas	Lung	VATS<br>Partial lung resection<br>Chemotherapy (cisplatin and irinotecan)	Died after 3 months-large cell carcinoma had metastasized to both lungs	[28]
**Genitourinary**							
Vincent T.H.B.M. Smit et al., 1988	68 F<br>Lower abdominal pain, abdominal distention	Laparotomy=a cystic mass on each ovary and multiple small tumors in parietal peritoneum and omentum majus and on the sigmoid's serosa	Both ovarian tumors=poorly differentiated mixed epithelial carcinoma, grade III<br>Uterine malignant mesodermal mixed tumor	Ovaries<br>Uterus	Total histerectomy<br>Bilateral salphingo-oophorectomy<br>Total omentectomy<br>Chemotherapy(Adriamycin+Alkeran)	Not specified	[29]

Liang Song et al., 2018	63 F<br>Vaginal fluid discharge and abdominal pain	Pelvic examination=cervical papillary hyperplasia<br>CT=adnexal masses with irregular shapes and bilateral mass fusion<br>Enhanced CT=bilateral "ovarian pedicle" sign	Endometrial poorly differentiated serous adenocarcinoma<br>Poorly differentiated squamous cell carcinoma of the cervix<br>Left-sided fallopian tube carcinoma	Uterus<br>Cervix<br>Fallopian tube	Total abdominal histerectomy<br>Bilateral salpingo-oophorectomy<br>8 courses of adjuvant chemotherapy(taxane carboplatin/ taxane cisplatin)	Alive, 18 months	[30]
Hirofumi Kurose et al., 2020	78 M<br>Chronic pancreatitis<br>Chronic smoker	CT=tumor in the right kidney,	Clear cell carcinoma<br>Adenocarcinoma of prostate<br>Urothelial carcinoma of urinary bladder	Kidney<br>Prostate<br>Urinary bladder	Partial nephrectomy<br>Transurethral resection of bladder tumor<br>Hormonal therapy for PC-Degarelix<br>Chemotherapy (Nivolumab/ Ipilimumab)	Alive, 1 year	[31]
Ahmed Abu-Zaid et al., 2017	55 F<br>Pelvic/abdominal mass for 2 months, abdominal distention, occasional vaginal bleeding	US=15 cm pelvic mass at the left adnexal site<br>CT=heterogeneous 14x12 cm pelvic/abdominal mass	Endometroid adenocarcinoma<br>Clear-cell carcinoma of the left ovary<br>Poorly differentiated squamous cell carcinoma of the cervix	Uterus<br>Ovary<br>Cervix	Abdominal histerectomy<br>Bilateral salpingo-oophorectomy<br>Infracolic omentectomy<br>Chemotherapy (paclitaxel/carboplatin)<br>Radiation therapy	Not specified-Recurrence after 3 months	[32]
Laura Gutierez-Palomino et al., 2015	49 F<br>History of ovarian endometriosis	Endometrial biopsy by aspiration for metrorrhagia	Endometrial adenocarcinoma<br>Endometrioid ovarian adenocarcinoma<br>Endometrioid tube adenocarcinoma	Uterus<br>Ovary<br>Fallopian tube	Omenectomy<br>Histerectomy<br>Appendectomy<br>Adjuvant chemotherapy (taxol and cisplatin)<br>Radiotherapy	Not specified	[33]
Kyler W. Perry et al., 2023	57 M<br>Dyspnea and chest pain on exertion, fatigue, hematuria for one year	CT=multi-lobulated enhancing mass in the bladder and a heterogeneous mass in the left renal calyx	Acinar adenocarcinoma of the prostate<br>Chromophobe renal cell carcinoma<br>Urothelial carcinoma	Bladder<br>Prostate<br>Kidney	Open radical cystoprostatectomy<br>Left laparoscopic radical nephrectomy<br>Adrenalectomy	Alive, 1.5 years	[34]
Vorapong Phupong et al., 2007	50 F<br>Menorrhagia for 2 mo<br>Asymptomatic pelvic mass for 3 years	US= myomatous masses in the uterus and cervix, echogenic ovarian cyst<br>Pap smear=negative	Adenosquamous carcinoma of the endocervix<br>Adenocarcinoma of the endometrium<br>Mucinous cystadenocarcinoma of the left ovary	Cervix<br>Uterus<br>Ovary	Subtotal histerectomy<br>Bilateral salpingo-oophroectomy<br>Omentectomy	Died- 3 months-pulmonary embolism	[35]
Eriko Takatori et al., 2014	50 F<br>Metrorrhagia	MRI=thickening of the endometrium and enlarged left ovary	Serous ovarian adenocarcinoma<br>Low-grade endometrioid adenocarcinoma<br>Endocervical mucinous adenocarcinoma	Ovary<br>Uterus<br>cervix<br>C	Radical histerectomy<br>Chemotherapy (paclitaxel/carboplatin)	Alive, 1.5 years	[36]
Takada T et al., 2002	72 M<br>Asymptomatic hematuria	Cystourethroscopy= a papillary bladder tumor<br>CT and MRI=mass lesion in the left kidney<br>Angyography= hypervascular lesion in the left kidney	Renal cell carcinoma<br>Transitional cell carcinoma of the urinary bladder<br>Moderately differentiated adenocarcinoma of the prostate	Kidney<br>Urinary bladder<br>Prostate	Total nephroureterectomy<br>Hilar lymphadenectomy<br>Transurethral rection of the bladder tumor	Not specified	[37]
Vallejo Herrador J. et al., 2002	59 M<br>Intermittent episodes of asymptomatic hematuria and acute urinary retention	Digital rectal examination= enlarged prostate<br>CT=intravesical lesion, an inhomogeneous mass of solid appearance in the kidney and left renal parahilar lesion	Urothelial carcinoma<br>Renal multifocal adenocarcinoma<br>Uothelial carcinoma	Urethra<br>Kidney	Transurethral resection of the bladder formation<br>Left radical and regional lymphadenectomy<br>radical cystoprostatectomy	Alive, 5 years	[38]
Li-Chuan Hsu et al., 2020	42 F<br>Enlarging pelvic mass<br>Low back pain	Abdominal CT = multiple mural myomas with obstruction at the cervix	Cervical carcinosarcoma<br>Endometrial endometrioid carcinoma<br>Ovarian mucinous carcinoma	Cervix<br>Uterus<br>Ovary	Abdominal total hysterectomy<br>Bilateral salpingo-oophorectomy<br>Bilateral pelvic lymphadenectomy<br>Partial omentectomy	Alive, 16 months	[39]
Deng F et al., 2022	81 M<br>Penile lesion	Cytoscopy= a 1.5 cm tumor in the bladder wall and a firm prostate in bilateral lobes	Penile squamous cell carcinoma<br>Bladder papillary urothelial carcinoma<br>Prostate adenocarcinoma	Penis<br>Urinary bladder<br>Prostate	Transurethral resection of the bladder	Not specified	[40]
Ogawa S et al., 2012	67 M<br>History of asymptomatic gross hematuria	Cytoscopy= nonpapillary sessile tumor and papillary pedunculated tumor on the blader	Bladder small cell carcinoma<br>Prostatic ductal adenocarcinoma	Urinary bladder<br>Prostate<br>Penis	Laparoscopic radical cystectomy<br>Urethrectomy<br>Partial penectomy	Alive, 6 months	[41]

			Penile squamous cell carcinoma		Chemotherapy (cisplatin+ irinotecan)		
Terada T. et al., 2010	73 M<br>Hematuria and lumbago	CT, MRI= bladder, ureter and kidney tumors	Polyploid tumor of the urinary blader -consisting of pleomorphic sarcomatoid carcinoma (80%) and high-grade papillary urothelial carcinoma (20%)<br>Renal squamous cell carcinoma	Kidney<br>Urinary bladder<br>Ureter	Cystectomy<br>Left nephroureterectomy	Alive, 3 months	[42]
**Head and Neck**							
Luisa Bresadola et al., 2021	68 M<br>History of heavy smoking and alcohol intake		Squamous cell carcinoma of the soft palate<br>Large ulcero-infiltrative invasive moderately/ poorly differentiated SCC of the tongue<br>Invasive well-differentiated SCC of the pharynx	Soft palate<br>Tongue<br>Pharynx	Left tongue hemiglossectomy with neck dissection<br>No adjuvant postoperative radiotherapy	Died- less than 6 months	[43]
Kenko Cupisti et al., 2005	72 M<br>Nonspecific neck pain<br>No family history of any endocrinopathy<br>Heavy smoker, obese, with an ischemic heart disease, hypertension and diabetes mellitus	CT=a left thyroid lobe nodule<br>US=a multinodular goiter with a dominant nodule on the left lobe<br>Fine-needle aspiration= a nodular thyroid gland with a follicular lesion	Papillary microcarcinoma in the right lobe<br>Follicular carcinoma of the right lobe<br>Left lobe medullary carcinoma<br>Diffuse and nodular C-cell hyperplasia	Thyroid	Total thyroidectomy	Alive, 3 months	[44]
Evangelos Giotakis et al., 2020	42 M<br>2 months history of a right level II lymphadenopath y<br>Heavy smoker	ENT examination= normal<br>18-FDG PET CT=elevated hypermetabolic of the lymph nodes, on both pharyngeal tonsils, in the base of the tongue and in the nasopharynx	Invasive SCC of the right tonsil<br>Invasive SCC of the tongue base<br>In situ carcinoma of the nasopharynx	Right tonsil<br>Tongue<br>Nasophary nx	Intensity modulated RT (IMRT) and cisplatin-based chemotherapy	Not specified	[45]
Jakub Piatkowsc ki et al., 2022	48 F<br>6 months history of slowly growing masses located bilaterally in the parotid region<br>Normal facial nerve function	MRI= two lesions in the superficial and deep lobes of the right parotid gland and one lesion in the superficial lobe of the left parotid gland	Basal cell adenomas	Parotid glands	Total parotidectomy with facial nerve preservation- on the right side<br>Superficial parotidectomy on the left side –after 6 months	Alive, 5 years	[46]
Andrew M. Plata et al., 2019	81 M<br>Dysphagia and pain in the right ear<br>65 years prior he underwent treatment of chronic sinusitis with nasopharyngeal radium irradiation	Esophagogastroscopy= normal<br>Otorhinolaryngology pan endoscopy= 3 separate tumors in the Waldeyer's ring<br>PET= PET-positive lesion on the base of the tongue and the nasopharynx, PET-negative area of lymphatic tag located in the palatopharyngeal mucosa	Mantle cell lymphoma of the torus tubarius<br>Posterior nasopharynx mantle cell lymphomas<br>Base of the tongue mantle cell lymphoma	Right Eustachian tube<br>Nasophary nx	-	Not specified	[47]
Fabio Bertone et al., 2021	66 M<br>Pharyngodynia<br>Clinical history of chronic alcohol-related liver disease and tobacco abuse	Endoscopic exam= 3 different lesions- on the left tonsil, on the epiglottis lingual surface and one on the right aryepiglottic fold	Squamous cell carcinoma	Left tonsil<br>Epiglottis<br>Right aryepiglotti c fold	CO2 laser left tonsillectomy<br>CO2 laser excision of the lesion on epiglottis free edge<br>CO2 laser excision of the right aryepiglottic fold lesion<br>Bilateral neck dissection and protective tracheostomy<br>Adjuvant radiotherapy	Alive, 6 months	[48]
Singh N.J. et al., 2015	71 M<br>Change in voice for 6 months<br>Respiratory difficulty	Laryngoscopy= ulcerative growth involving the left true and false vocal cords, anterior commissure and right true vocal cord<br>CECT=multiple soft tissue lesions involving the left hemi-larynx with erosion of the thyroid cartilage and spread to the strap muscles	Invasive keratinizing squamous cell carcinoma<br>Nodal marginal zone lymphoma<br>Papillary thyroid carcinoma	Thyroid<br>Larynx	Total laryngectomy<br>Total thyroidectomy<br>Bilateral selective neck dissection	Alive, 1 year	[49]
**Skin**							

De Giorgi V. et al., 2007	43 F<br>A pigmented lesion became asymmetric, with irregular borders and pigmentation	Dermatoscopy = suspected melanoma diagnosis for three pigmented lesions	Superficial spreading melanoma for all three lesions	Skin	Excisional biopsy	Not specified	[50]
**Different Systems**							
Dan Li et al., 2020	66 M<br>Progressive dysphagia	CT=thickening of the esophageal wall+lumen stenosis+ blurred peripheral fat gap<br>CT=1.9x1.2cm soft tissue mass shadow in the left upper lobe<br>MRI=massive shadow in the right anterior superior segment of the liver	Esophageal squamous cell carcinoma (moderate-to-poorly differentiated)<br>Lung adenocarcinoma (moderate-to-poorly differentiated)<br>Hepatocellular carcinoma (moderately differentiated)	Esophagus<br>Lung<br>Liver	Excision of left thoracic esophageal tumor<br>Wedge resection of left upper lobe<br>Liver tumor resection	Died after 1 year- respiratory failure due to pulmonary infection	[51]
Q. Huang et al., 2018	55 M<br>RU quadrant pain + palpable mass<br>Fluid stools	Colonoscopy = Hepatic flexure mass<br>CT = liver lesions and hydronephrosis of the right kidney (a large lymph node was obstructing the ureter)	Adenocarcinoma of the colon<br>Urothelial carcinoma<br>Malignant mesothelioma	Colon<br>Kidney<br>Peritoneum	Right hemicolectomy with right nephrectomy and wedge resection of the duodenum<br>ChT for the hepatic lesions	Not specified	[52]
T. Hayashi et al. 1996	66 M<br>Dysphagia<br>Sore throat<br>Smoked 20 cigs/day for 46 years, 5 cups of sake a day for 46 years	Endoscopy = two soft palate tumors, one in the epiglottis<br>Barium esophagography = tumor in the middle 1/3 of the esophagus	Poorly differentiated squamous cell carcinoma of the soft palate<br>Well differentiated squamous cell carcinoma of the larynx<br>Moderately differentiated squamous cell carcinoma of the esophagus	Soft palate<br>Larynx<br>Esophagus	Cht = cisplatin<br>Radiotherapy	Not specified	[53]
H. Ozan et al. 2008	52 F<br>Menometrorrhag ia	Gynecological examination = mass occupying the pelvis<br>US = mass that pushed the bladder anteriorly<br>Cystoscopy<br>Laparotomy = mass in the right adnexal area with omental adhesions, gallbladder filled with stones, with a nodule covering its neck	Ovarian endometrioid carcinoma with a clear cell component<br>Endometrial endometrioid adenocarcinoma<br>Mucinous adenocarcinoma of the gallbladder	Ovary<br>Uterus<br>Gallbladde r	Total abdominal hysterectomy with bilateral salpingo-oophorectomy<br>Bilateral pelvic and paraaortic lymph node dissection<br>Partial omentectomy<br>Cholecystectomy<br>6 cycles of cisplatin, Adriamycin, cyclophosphamide	Died, 16 months	[54]
E. Okajima et al. 1994	75 M	Routine US = right kidney and liver masses<br>Oral examination = oral floor mass	Renal cell carcinoma<br>Hepatocellular carcinoma<br>Squamous cell carcinoma of the oral floor	Kidney<br>Liver<br>Oral floor	Radical nephrectomy<br>Enucleation of the liver tumor<br>Resection of the tumor of the oral floor	Not specified	[55]
F. Iqbal et al. 2008	71 M<br>One-month history of hoarseness and shortness of breath on exertion	CT = tumour involving both vocal cords and eroding the thyroid cartilage<br>Bronchoscopy = lesion in the left bronchus	Well differentiated squamous cell carcinoma of the larynx<br>Sclerosing papillary carcinoma of the thyroid<br>Left bronchus adenocarcinoma	Larynx<br>Thyroid<br>Bronchus	Left hemithyroidectomy<br>Total laryngectomy	Died after 1 month, multi-organ failure	[56]
E. Mittra et al., 2007	61 M<br>Weight loss<br>Dysphagia	F-18 FDG PET = 3 synchronous primary malignancies	Squamous cell carcinoma of the midesophagus<br>Adenocarcinoma of the right lung<br>Squamous cell carcinoma of the left tonsil	Esophagus<br>Tonsil<br>Lung	Cisplatin/5-FU<br>External beam radiotherapy to the esophageal mass<br>Tonsillectomy	Not specified	[57]
W. Makis et al., 2011	66 F<br>Diarrhea<br>Weight loss	F-18 FDG PET/CT = 3 masses – breast, rectum, urinary bladder<br>Follow-up PET/CT: liver and lung metastases	Moderately differentiated adenocarcinoma of the rectum<br>Invasive ductal carcinoma of the right breast<br>High-grade urothelial carcinoma	Rectum<br>Breast<br>Urinaryy bladder	Palliative radiation therapy<br>Anastrozole<br>Bladder curettage<br>Intravesical BCG	Not specified	[58]
M.E. Căpîlna et al., 2015	61 F<br>Vaginal bleeding	US = thickened endometrium, right ovarian mass<br>CT = right ovarian mass<br>Uterine curettage = endometroid adenocarcinoma of the endometrium<br>Laparatomy = right adnexal tumour involving	High-grade serous adenocarcinoma of right adnexa<br>Endometroid adenocarcinoma of the endometrium with mucosal invasion<br>Moderately differentiated adenocarcinoma of the recto-sigmoid junction	Colorectu m<br>Ovary<br>Uterus	Total abdominal hysterectomy<br>Bilateral adnexectomy<br>Total omentectomy<br>Appendectomy<br>Recto-sigmoid colon resection	Alive, 3 months	[59]

		both tube and ovary,<br>sigmoid colon tumor					
A Nishikawa et al., 2014	82 F<br>Breast masses	Physical examination = 2 masses on the left chest wall<br>Mammography, US = breast tumor<br>F-18 FDG PET/CT = focal accumulation in the lower rectum<br>Colonoscopy = rectal carcinoma	Diffuse large B-cell lymphoma of the breast<br>Invasive lobular carcinoma of the breast<br>Well- to moderately differentiated adenocarcinoma of the rectum	Breast<br>Rectum	Left mastectomy with axillary lymph node dissection<br>Adjuvant Anastrozole<br>Hartmann's operation	Alive, 7 months	[60]
G. E. Ringler et al., 1989	60 F<br>Abdominal swelling and pain	FDG PET scan = left tonsillar uptake<br>Endoscopy= esophageal tumor<br>Previously diagnosed with 2 synchronous malignancies	Well-differentiated mucinous cystadenocarcinoma of the left ovary<br>Granular cell carcinoma of the right kidney<br>Well-differentiated adenocarcinoma of the right lung	Ovary<br>Kidney<br>Lung	Hysterectomy with bilateral salpingo-oophorectomy<br>Right nephrectomy<br>Right upper lobectomy	Not specified	[61]
M. S. Eren et al., 2012	58 F<br>Uterine bleeding	Fractionated curettage = endometrial cancer<br>PET/CT = thyroid gland and left lung pathologic uptake<br>Neck US = Thyroid nodule	Endometrial adenocarcinoma<br>Peripheral carcinoid tumor of the left lung<br>Papillary carcinoma of the thyroid	Thyroid<br>Uterus<br>Lung	Total abdominal hysterectomy<br>Bilateral salpingo-oophorectomy	Not specified	[62]
S. Patel et al., 1985	71 F<br>Epigastric distress	Physical examination = asymptomatic right breast mass<br>Gastroscopy = stomach lesion<br>X-ray = density in the right upper lung	Squamous cell carcinoma of the right lung<br>Infiltrating ductal carcinoma of the right breast<br>Adenocarcinoma of the stomach	Stomach<br>Lung<br>Breast	Right pulmonary lobectomy<br>Right radical mastectomy<br>Wide-field hemigastrectomy	Alive, 2 years	[63]
H. Katz et al., 2017	48 F<br>Lower abdominal pain	Physical exam = large abdominal mass reaching to the umbilicus<br>CT = abdominal mass in the RUQ and one in the pelvis	Granulosa cell tumor of the right ovary<br>Right adrenocortical carcinoma<br>Moderately differentiated adenocarcinoma of the sigmoid colon	Ovary<br>Adrenal gland<br>Colon	Pelvic mass resection<br>Total abdominal hysterectomy<br>Bilateral salpingo-oophorectomy<br>Bilateral pelvic lymphadenectomy and omentectomy<br>Right radical adrenalectomy<br>Sigmoidectomy	Alive, 2 years	[64]
L. Zhi-Ke et al., 2022	64 M<br>Hematuria<br>Dysuresia	CT/US = tumors in the bladder triangle area and prostate<br>CT = nodule located in the left lung	Bladder urothelial carcinoma<br>Adenocarcinoma of the left lung<br>Prostate adenocarcinoma	Urinary bladder<br>Lung<br>Prostate	Bladder intravesical chemotherapy – gemcitabine<br>Radical cystoprostatectomy<br>4 cycles of pemetrexed and carboplatin plus pembrolizumab (+ bevacizumab from the 5th cycle)<br>Left lung radiotherapy	Alive, 2 years	[65]
S. Kurul et al., 2013	56 F<br>Hyperpigmented mass in left leg	18F-FDG PET/CT = left breast mass, abnormal lymph node uptake in the left pelvis region, abnormal uptake on the lower lobe of right lung	Malignant melanoma<br>Non-small cell lung cancer<br>Invasive ductal carcinoma of the left breast	Skin<br>Lung<br>Breast	Stereotactic body radiotherapy<br>Subcutaneous nipple sparing mastectomy<br>Six cycles of cyclophosphamide, epirubicin and 5-flurouracil based chemotherapy and trastzumab, anastrozole	Alive, 1.5 years	[66]
S. Kataoka et al., 2017	72 M<br>Nausea<br>Heartburn	Gastroscopy = 2 esophageal tumors, one pharyngeal tumor	Esophageal squamous cell carcinoma<br>Well differentiated esophageal adenocarcinoma<br>Pharyngeal squamous cell carcinoma	Esophagus<br>Pharynx	Neoadjuvant chemotherapy – 5-FU, CDDP<br>Minimally invasive esophagectomy<br>Endoscopic submucosal dissection for the pharyngeal tumor	Alive, 4 years	[67]
L. Peng et al., 2018	32 F	CT = mass in the right lobe of the thyroid and the left upper kidney, nodule in the left lower lobe of the lung	Renal clear cell carcinoma<br>Thyroid papillary carcinoma<br>Moderately differentiated lung adenocarcinoma	Kidney<br>Thyroid<br>Lung	Partial nephrectomy of the left kidney<br>Right thyroidectomy<br>Right upper lobectomy of the lung<br>4 cycles of adjuvant chemotherapy with pemetrexed/cisplatin	Not specified	[68]
O. Oey et al., 2023	51 M<br>Hematuria<br>Dysuria<br>Prior prostate cancer, treated with prostatectomy	CT = a hepatic lesion, left bladder wall lesion, enlarged perigastric lymph nodes<br>Gastroscopy = gastric lesion	High-grade urothelial carcinoma<br>Papillary thyroid carcinoma<br>Gastric neuroendocrine tumor	Urinary bladder<br>Thyroid<br>Stomach	Cht with 5-FU and mitomycin<br>Total thyroidectomy<br>Somatostatin analogue treatment (for the NET)	Alive, 9 months	[69]

		PET = thyroid tumor suspicion<br>Cystoscopy<br>Gallium octreotide PET = hepatic lesions, pancreatic lesion – metastases from the gastric NET?					
S. J. Oh et al., 2015	50 M	Routine upper endoscopy = gastric cancer<br>CT = left renal mass<br>US = thyroid nodules	Renal clear cell carcinoma<br>Poorly differentiated adenocarcinoma of the stomach<br>Papillary thyroid carcinoma	Stomach<br>Kidney<br>Thyroid	Radical subtotal gastrectomy<br>Left partial nephrectomy<br>Radical thyroidectomy	Not specified	[70]
B. Qiu et al., 2022	59 M<br>Fever<br>Night sweats<br>Weight loss	CT = rectum, liver lesions and spleen nodules<br>Lab = Hep. B infection	Diffuse large B-cell lymphoma<br>Moderately differentiated rectal adenocarcinoma<br>Hepatocellular carcinoma	Lymphatic<br>Rectum<br>Liver	Chemotherapy with R-Gemox (for DLBCL)<br>R-CHOP + sintilimab (targeting HCC) => cure of DLBCL and PR of HCC<br>Laparoscopic Dixon operation for the rectal cancer<br>Radical right hemihepatectomy<br>Percutaneous microwave ablation for local recurrence	Alive, 6 months	[71]
X. Song et al., 2016	63 F<br>Dysphagia	Endoscopy = esophageal mass<br>CT = right upper lobe lung lesion, mediastinal mass	Poorly differentiated esophageal squamous cell carcinoma<br>Invasive adenocarcinoma of the right lung<br>Thymoma	Thymus<br>Esophagus<br>Lung	Esophagectomy<br>Lobectomy<br>Thymomectomy	Alive, 6 months	[72]
J. S. Lee et al., 2010	56 F<br>Hematochezia	Colonoscopy = rectal mass<br>PET-CT = rectal mass, left thyroid lobe nodule<br>Pap smear = cervical cancer	Mucinous adenocarcinoma with signet-ring cells of the rectum<br>Papillary carcinoma of the thyroid gland<br>Squamous cell carcinoma of the uterine cervix	Uterus<br>Thyroid gland<br>Rectum	Palliative chemotherapy with FOLFOX	Died, 1 year	[73]
L. E. Mendez et al., 2016	60 F<br>Abdominal pain<br>Flatulence<br>Bloating<br>Diarrhea<br>Postmenopausal bleeding	Endometrial biopsy = adenocarcinoma<br>PET = right upper pole renal mass<br>Colonoscopy = transverse colon lesion	Moderately differentiated endometrial adenocarcinoma<br>Clear cell carcinoma of the right kidney<br>Poorly differentiated mucinous adenocarcinoma with signet ring cell of the transverse colon	Colon<br>Uterus<br>Kidney	Total abdominal hysterectomy<br>Bilateral salpingo-oophorectomy<br>Laparoscopic right nephrectomy<br>Right transverse colectomy<br>Cht + radiation therapy	Alive, 2 years	[74]
R. Huang et al., 2022	61 M<br>Dull pain in the lower abdomen<br>Mild fever<br>Loss of appetite	CT = small intestine wall thickening<br>18F-FDG = uptake in the lingula of the left lung and the left bladder wall	Urothelial carcinoma of the bladder<br>Diffuse large B-cell lymphoma<br>Well to moderately differentiated squamous cell carcinoma of the left lung	Bladder<br>Lung<br>Lymphatic	Transurethral resection of bladder tumor<br>R-CHOP<br>Left upper lobectomy	Alive, 3 months	[75]
C. L. Park et al., 2023	66 F	CT = bilateral pulmonary lesions<br>PET = lesions in the lungs and breasts<br>Mammogram = bilateral multifocal breast masses	Adenocarcinoma of the left lung<br>Hormone receptor-positive invasive ductal carcinoma of the left breast<br>Triple negative invasive ductal carcinoma of the right breast	Breast<br>Lung	Radiotherapy<br>Osimertinib + carboplatin/nab-paclitaxel<br>Doxorubicin + cyclophosphamide<br>Right mastectomy<br>Letrozole with osimertinib	Alive, 20 months	[76]
E. I. Ágoston et al., 2018	70 F	Mammography = left breast mass with axillary lymph nodes metastasis<br>Colonoscopy = tumor in the ascending colon<br>CT = left kidney mass	Invasive left breast carcinoma with low-grade ductal carcinoma in situ<br>Moderately differentiated mucinous adenocarcinoma of the ascending colon<br>Renal cell carcinoma of the left kidney	Breast<br>Kidney<br>Colon	Right hemicolectomy<br>Left nephrectomy<br>Aromatase inhibitor therapy<br>Left sector excision and axillary block dissection<br>Adjuvant 5-fluorouracil, epiadrimicin, cyclophosphamide chemotherapy<br>Radiotherapy	Alive, 16 months	[77]
R. S. Rai et al., 2007	79 M<br>Hematuria<br>Right hip pain<br>Neck swellings	Rectal examination = enlarged prostate<br>US/CT = left kidney lesion	Renal cell carcinoma of the left kidney<br>Prostate adenocarcinoma<br>Thyroid follicular carcinoma	Prostate<br>Kidney<br>Thyroid	Left radical nephrectomy<br>Near-total thyroidectomy	Alive, 2 years	[78]
E. Lee et al., 2018	63 F<br>Vaginal bleeding	US = polypoid mass in the endometrium<br>Pap smear = presence of atypical glandular cells<br>CT = endometrial cancer suspicion, right breast lesion	Carcinosarcoma of the uterus<br>Invasive ductal carcinoma of the right breast	Uterus<br>Colon<br>Breast	Total abdominal hysterectomy<br>Bilateral salpingo-oophorectomy<br>Omentectomy<br>Segmental resection of descending colon<br>Right radical mastectomy	Not specified	[79]

		Colonoscopy = descending colon malignancy			Radiotherapy<br>Paclitaxel + carboplatin		
K. H. AlBaqmi et al., 2020	63 M<br>Abdominal pain<br>Constipation	PE = distended abdomen<br>CT = large bowel obstruction at the splenic flexure, left kidney lesion<br>Laparotomy = stomach mass at the greater curvature	Papillary renal cell carcinoma<br>Moderately differentiated colonic adenocarcinoma<br>Gastrointestinal stromal tumor	Stomach<br>Kidney<br>Colon	Laparotomy = colon and kidney masses excision, wedge resection of the stomach mass	Not specified	[80]
C. J. Jin et al., 2015	66 F	Mammography = left breast abnormality<br>CT = bilateral lung masses	Moderately differentiated infiltrating ductal cancer of the left breast<br>Bilateral acinar type adenocarcinoma of the lungs	Breast<br>Lung	Left partial mastectomy<br>Radiation therapy<br>Letrozole	Alive, 6 months	[81]
S. Y. Jeon et al., 2008	74 M	X-ray = chest nodule<br>CT = nodule in the left upper lobe of the lung<br>FDG-PET/CT + Endoscopy = esophageal and laryngeal tumors	Adenocarcinoma of the left lung<br>Squamous cell carcinoma of the esophagus and larynx	Lung<br>Esophagus<br>Larynx	Left upper lobectomy<br>Paclitaxel + carboplatin<br>Radiation therapy	Alive, 2 years	[82]

References:

1. Allan A. Triple synchronous colorectal carcinoma causing intestinal obstruction. Bristol Med Chir J. 1983 Oct;98(368):170-2

2. Schmid KW, Glaser K, Wykypiel H, Feichtinger H. Synchronous adenocarcinoma of the transverse colon, the gallbladder and the vermiform appendix. Klin Wochenschr. 1988 Nov 1;66(21):1093-6. doi: 10.1007/BF01711925

3. Sauri FM, Zakarneh EA, Alessa MY, Sakr AH, Chung YS, Kim HS, Kim NK. Triple synchronous primary neoplasms in the gastrointestinal tract. Chin Med J (Engl). 2021 Feb 3;134(10):1233-1235. doi: 10.1097/CM9.0000000000001389

4. Yeh CC, Hsi SC, Chuu CP, Kao YH. Synchronous triple carcinoma of the colon and rectum. World J Surg Oncol. 2013 Mar 13;11:66. doi: 10.1186/1477-7819-11-66

5. Jiang W, Zhang G, Li H, Xu X, Jia L, Luo X, Cao Z. Synchronous triple primary gastrointestinal malignant tumors treated with laparoscopic surgery: A case report. Open Med (Wars). 2023 Jul 3;18(1):20230742. doi: 10.1515/med-2023-0742

6. Boland BM, Collins CG, Christiansen E, O'Brien A, Duignan J. Three synchronous gastrointestinal tumours. Ir J Med Sci. 2011 Dec;180(4):897-900. doi: 10.1007/s11845-009-0295-7. Epub 2009 Mar 17

7. De Rosa M, Rondelli F, Stella P, Boni M, Ermili F, Ceccarelli G. Triple synchronous colorectal carcinoma. ANZ J Surg. 2019 Jul;89(7-8):E329-E330. doi: 10.1111/ans.14452

8. Cheng J, Liu X, Shuai X, Deng M, Gao J, Tao K. Synchronous triple colorectal carcinoma: a case report and review of literature. Int J Clin Exp Pathol. 2015 Aug 1;8(8):9706-11

9. Petroianu A, Sabino KR, Nunes MB. Synchronous triple squamous cell carcinoma of the esophagus. Int J Surg Case Rep. 2018;49:34-36. doi: 10.1016/j.ijscr.2018.05.028

10. Wang P, Zhang G, Shen H. Multiple synchronous primary malignancies induced by benzene exposure: a case report. J Occup Med Toxicol. 2009 Apr 16;4:7. doi: 10.1186/1745-6673-4-7

11. Zhan X, He L, Song K, Cao S, Meng E, Wang Y. Case Report: Triple Primary Malignant Tumors of the Esophagus, Stomach, and Colon in a Patient With Genetic Analysis. Front Genet. 2021 Jul 9;12:676497. doi: 10.3389/fgene.2021.676497

12. Aloraini AM, Helmi HA, Aljomah NA, Zubaidi AM. Multiple primary gastrointestinal tumors of gastric, pancreatic and rectal origin; a case report. Int J Surg Case Rep. 2021 Dec;89:106610. doi: 10.1016/j.ijscr.2021.106610

13. Chong VH, Idros A, Telisinghe PU. Triple synchronous gastrointestinal malignancies: a rare occurrence. Singapore Med J. 2010 Oct;51(10):e176-7

14. Fukaya M, Abe T, Yokoyama Y, Itatsu K, Nagino M. Two-stage operation for synchronous triple primary cancer of the esophagus, stomach, and ampulla of Vater: report of a case. Surg Today. 2014 May;44(5):967-71. doi: 10.1007/s00595-013-0549-x

15. Tamura M, Shinagawa M, Funaki Y. Synchronous triple early cancers occurring in the stomach, colon and gallbladder. Asian J Surg. 2003 Jan;26(1):46-8; discussion 49. doi: 10.1016/S1015-9584(09)60216-5
16. Guoliang S, Dongsheng H. Triple synchronous malignant tumors of colon, appendix and liver: A case report with literature review. Pak J Med Sci. 2013 Jan;29(1):237-8. doi: 10.12669/pjms.291.2277
17. Ohtsubo K, Ishikawa D, Nanjo S, Takeuchi S, Yamada T, Mouri H, Yamashita K, Yasumoto K, Gabata T, Matsui O, Ikeda H, Takamatsu Y, Iwakami S, Yano S. Synchronous triple cancers of the pancreas, stomach, and cecum treated with S-1 followed by pancrelipase treatment of pancreatic exocrine insufficiency. JOP. 2013 Sep 10;14(5):515-20. doi: 10.6092/1590-8577/1719
18. Sato K, Maekawa T, Yabuki K, Tamasaki Y, Maekawa H, Kudo K, Sengoku H, Kawa I, Wada R, Matsumoto M. A case of triple synchronous cancers occurring in the gallbladder, common bile duct, and pancreas. J Gastroenterol. 2003;38(1):97-100. doi: 10.1007/s005350300014
19. Leong BD, Ramu P, Kumar VM, Chuah JA. Synchronous adenocarcinoma of caecum, transverse colon and jejunum. Med J Malaysia. 2008 Jun;63(2):148-9
20. Chen JH, Chen CC, Tzeng LM, Tsay SH, Chiang JH, Lu CC, Chang FY, Lee SD. Resection of triple synchronous tumors--gastric adenocarcinoma, gallbladder adenocarcinoma and stromal tumor of the stomach. Zhonghua Yi Xue Za Zhi (Taipei). 2001 Nov;64(11):655-60
21. Omori K, Nomoto Y, Kawamura T, Kubooka M, Kawaguchi K, Ii N, Takada A, Toyomasu Y, Sakuma H. Endobronchial brachytherapy combined with surgical procedure for synchronous multiple primary lung cancer: A case report. Thorac Cancer. 2021 Apr;12(8):1252-1255. doi: 10.1111/1759-7714.13911
22. Kashif M, Ayyadurai P, Thanha L, Khaja M. Triple synchronous primary lung cancer: a case report and review of the literature. J Med Case Rep. 2017 Sep 1;11(1):245. doi: 10.1186/s13256-017-1410-4
23. Yoon HJ, Lee HY, Han J, Choi YL. Synchronous triple primary lung cancers: a case report. Korean J Radiol. 2014 Sep-Oct;15(5):646-50. doi: 10.3348/kjr.2014.15.5.646. Epub 2014 Sep 12. PMID: 25246827; PMCID: PMC4170167.
24. Koyama T, Shimizu K, Uehara T, Matsuoka S, Takeda T, Yamada K, Eguchi T, Hamanaka K, Sano K. Synchronous triple primary lung cancer with three different histological subtypes in the same lobe: A case report. Thorac Cancer. 2021 Mar;12(5):711-714. doi: 10.1111/1759-7714.13796
25. Gorospe L, Gómez-Bermejo MÁ, Paredes-Rodríguez P, Mirambeaux-Villalona RM, Fra-Fernández S, Muñoz-Molina GM, Benito-Berlinches A. Three synchronous Lung Cancers in the Same Lung Segment: Triple Trouble? Arch Bronconeumol. 2023 Jul;59(7):458-460. doi: 10.1016/j.arbres.2023.02.001
26. Kim EK, Kim JY, Kim BM, Lim SN. Multiple primary malignancies of laryngeal cancer, small cell lung cancer and squamous cell lung cancer in a patient: how to approach MPMs. BMJ Case Rep. 2017 Mar 7;2017:bcr2016216305. doi: 10.1136/bcr-2016-216305
27. Okita R, Shimizu K, Mimura T, Miyata Y, Okada M, Arihiro K. Suggestive synchronous triple squamous cell carcinoma of the lung in the same lobe. Gen Thorac Cardiovasc Surg. 2010 Aug;58(8):427-30. doi: 10.1007/s11748-009-0476-3
28. Tokuchi Y, Kamachi M, Harada M, Hasegawa M, Mishina T, Yamashiro K, Suzuki H, Isobe H. Synchronous triple lung cancers after treatment for non-Hodgkin's lymphoma: metachronous quadruple cancers. Intern Med. 2003 Oct;42(10):1031-4. doi: 10.2169/internalmedicine.42.1031
29. Smit VT, Cornelisse CJ, De Jong D, Dijkshoorn NJ, Peters AA, Fleuren GJ. Analysis of tumor heterogeneity in a patient with synchronously occurring female genital tract malignancies by DNA flow cytometry, DNA fingerprinting, and immunohistochemistry. Cancer. 1988 Sep 15;62(6):1146-52. doi: 10.1002/1097-0142(19880915)62:6<1146::aid-cncr2820620618>3.0.co;2-d
30. Song L, Li Q, Yang K, Yin R, Wang D. Three primary synchronous malignancies of the uterus, cervix, and fallopian tube: A case report. Medicine (Baltimore). 2018 Jun;97(24):e11107. doi: 10.1097/MD.0000000000011107
31. Kurose H, Ueda K, Nakiri M, Matsuo M, Suekane S, Igawa T. Synchronous primary triple urogenital malignant tumors of kidney, prostate and bladder. Urol Case Rep. 2020 May 25;33:101277. doi:

10.1016/j.eucr.2020.101277
32. Abu-Zaid A, Alsabban M, Abuzaid M, Alomar O, Salem H, Al-Badawi IA. Triple Synchronous Primary Neoplasms of the Cervix, Endometrium, and Ovary: A Rare Case Report and Summary of All the English PubMed-Indexed Literature. Case Rep Obstet Gynecol. 2017;2017:9705078. doi: 10.1155/2017/9705078
33. Gutiérrez-Palomino L, Romo-de Los Reyes JM, Pareja-Megía MJ, García-Mejido JA. Tumores triple sincrónicos ginecológicos. Reporte de un caso [Triple synchronous primary gynaecological tumours. A case report]. Cir Cir. 2016 Jan-Feb;84(1):69-72. doi: 10.1016/j.circir.2015.06.015
34. Perry KW, Yankelevich G, Ashton L, Diorio G. Triple Synchronous Urogenital Malignancies of the Bladder, Kidney, and Prostate: Management in a Single Operation. Cureus. 2023 Oct 16;15(10):e47107. doi: 10.7759/cureus.47107
35. Phupong V, Khemapech N, Triratanachat S. Triple synchronous primary cervical, endometrial and ovarian cancer with four different histologic patterns. Arch Gynecol Obstet. 2007 Dec;276(6):655-8. doi: 10.1007/s00404-007-0392-7
36. Takatori E, Shoji T, Miura Y, Takeuchi S, Uesugi N, Sugiyama T. Triple simultaneous primary invasive gynecological malignancies: a case report. J Obstet Gynaecol Res. 2014 Feb;40(2):627-31. doi: 10.1111/jog.12199
37. Takada T, Honda M, Momohara C, Komori K, Fujioka H. [Synchronous triple urogenital cancer (renal cancer, bladder cancer, prostatic cancer): a case report]. Hinyokika Kiyo. 2002 Apr;48(4):239-42
38. Vallejo Herrador J, Sánchez de la Muela P, Diz Rodríguez R, Martín-Laborda F. Triple neoplasia urológica primaria sincrónica. Aportación de un nuevo caso y revisión de la literatura [Synchronous primary urologic triple neoplasia. Report of a new case and review of the literature]. Actas Urol Esp. 2002 Jan;26(1):57-9. doi: 10.1016/s0210-4806(02)72731-9
39. Hsu LC, Chiang AJ. Triple synchronous malignancies of the female genital tract with an advanced stage carcinosarcoma of the uterine cervix: A case report. Taiwan J Obstet Gynecol. 2020 Jul;59(4):613-614. doi: 10.1016/j.tjog.2020.05.026
40. Deng F, Kong MX, Lai J. Synchronous Penile Squamous Cell Carcinoma, Bladder Urothelial Carcinoma and Prostate Adenocarcinoma Diagnosed in One Procedure. Anticancer Res. 2022 Jul;42(7):3601-3605. doi: 10.21873/anticanres
41. Ogawa S, Yasui T, Taguchi K, Umemoto Y, Kojima Y, Kohri K. The probability of involvement of human papillomavirus in the carcinogenesis of bladder small cell carcinoma, prostatic ductal adenocarcinoma, and penile squamous cell carcinoma: a case report. BMC Res Notes. 2014 Dec 15;7:909. doi: 10.1186/1756-0500-7-909
42. Terada T. Synchronous squamous cell carcinoma of the kidney, squamous cell carcinoma of the ureter, and sarcomatoid carcinoma of the urinary bladder: a case report. Pathol Res Pract. 2010 Jun 15;206(6):379-83. doi: 10.1016/j.prp.2009.07.021
43. Bresadola L, Weber D, Ritzel C, Löwer M, Bukur V, Akilli-Öztürk Ö, Becker J, Mehanna H, Schrörs B, Vascotto F, Sahin U, Kong A. Comprehensive Genomic and Transcriptomic Analysis of Three Synchronous Primary Tumours and a Recurrence from a Head and Neck Cancer Patient. Int J Mol Sci. 2021 Jul 15;22(14):7583. doi: 10.3390/ijms22147583
44. Cupisti K, Raffel A, Ramp U, Wolf A, Donner A, Krausch M, Eisenberger CF, Knoefel WT. Synchronous occurrence of a follicular, papillary and medullary thyroid carcinoma in a recurrent goiter. Endocr J. 2005 Apr;52(2):281-5. doi: 10.1507/endocrj.52.281
45. Giotakis E, Sakagiannis G, Delidis A, Maragkoudakis P, Economopoulou P, Zacharatou A, Kalkanis D, Psyrri A. Multiple synchronous squamous cell carcinomas of the head and neck: A case report. Oral Oncol. 2020 Oct;109:104717. doi: 10.1016/j.oraloncology.2020.104717
46. Piątkowski J, Garsta E, Śmigielski G, Markiet K, Wasąg B, Ciarka A, Mikaszewski B. Synchronous bilateral multifocal basal cell adenomas of the parotid gland-a case report. BMC Oral Health. 2022 Jul 29;22(1):314. doi: 10.1186/s12903-022-02339-3

47. Plata AM, Pollard RE, Fang Y, Khalid A, Estalilla OC, Jelic TM. Three Synchronous Primary Extranodal Mantle Cell Lymphomas Involving Torus Tubarius, Posterior Nasopharynx, and Base of the Tongue 65 Years After Treatment of Chronic Sinusitis with Nasopharyngeal Radium Irradiation. Am J Case Rep. 2019 Jul 21;20:1063-1070. doi: 10.12659/AJCR.915742
48. Bertone F, Robiolio E, Robiolio L, Liscia D, Gervasio CF. Three Synchronous Head and Neck Cancers: A Multidisciplinary and Surgical Challenge. Ear Nose Throat J. 2023 Jun;102(6):NP294-NP297. doi: 10.1177/01455613211007946
49. Singh NJ, Tripathy N, Roy P, Manikantan K, Arun P. Simultaneous Triple Primary Head and Neck Malignancies: A Rare Case Report. Head Neck Pathol. 2016 Jun;10(2):233-6. doi: 10.1007/s12105-015-0664-7
50. De Giorgi V, Salvini C, Sestini S, Vignoli M, Sestini R, Papi F, Lotti T. Triple synchronous cutaneous melanoma: a clinical, dermoscopic, and genetic case study. Dermatol Surg. 2007 Apr;33(4):488-91. doi: 10.1111/j.1524-4725.2007.33098.x
51. Li D, Yu M, Zhou P, Yang J, Wang Y. Whole-exome sequencing in a patient with synchronous triple primary malignancies involving lung cancer: a case report. Precis Clin Med. 2020 Jun 5;3(4):306-310. doi: 10.1093/pcmedi/pbaa019
52. Huang Q, He X, Qin H, Fan X, Xie M, Lian L. Triple primary malignancies in a patient with colorectal adenocarcinoma: A case report. Int J Surg Case Rep. 2018;42:34-37. doi: 10.1016/j.ijscr.2017.11.058
53. Hayashi T, Sagawa H, Kobuke K, Fujii K, Yokozaki H, Tahara E. Molecular-pathological analysis of a patient with three synchronous squamous cell carcinomas in the aerodigestive tract. Jpn J Clin Oncol. 1996 Oct;26(5):368-73. doi: 10.1093/oxfordjournals.jjco.a023247
54. Ozan H, Ozerkan K, Aker S, Bülbül M. A case with three primary tumors of the ovary, endometrium and gallbladder. Eur J Gynaecol Oncol. 2008;29(5):551-3
55. Okajima E, Ozono S, Nagayoshi J, Uemura H, Hirao Y, Nakajima Y, Nakano H, Yoshida M, Sugimura M, Okajima E. A case report of synchronous triple cancer resected simultaneously. Jpn J Clin Oncol. 1994 Jun;24(3):166-70
56. Iqbal FR, Sani A, Gendeh BS, Aireen I. Triple primary cancers of the larynx, lung and thyroid presenting in one patient. Med J Malaysia. 2008 Dec;63(5):417-8
57. Mittra E, Vasanawala M, Niederkohr R, Rodriguez C, Segall G. A case of three synchronous primary tumors demonstrated by F-18 FDG PET. Clin Nucl Med. 2007 Aug;32(8):666-7. doi: 10.1097/RLU.0b013e3180a1ad48
58. Makis W, Ciarallo A, Lisbona R. Three synchronous primary malignancies detected by F-18 FDG PET/CT: breast, rectal, and urothelial bladder carcinomas. Clin Nucl Med. 2011 Sep;36(9):791-4. doi: 10.1097/RLU.0b013e318217af6b
59. Căpîlna ME, Rusu SC, Laczko C, Szabo B, Marian C. Three synchronous primary pelvic cancers--a case report. Eur J Gynaecol Oncol. 2015;36(2):216-8
60. Nishikawa A, Kasai H, Koyama Y, Koide N, Iijima A, Shimojo H, Kumeda S. Synchronous ipsilateral carcinoma of the accessory mammary gland and primary lymphoma of the breast with subsequent rectal carcinoma: report of a case. World J Surg Oncol. 2014 Sep 14;12:286. doi: 10.1186/1477-7819-12-286
61. Ringler GE, Senekjian EK, Smith FL, Little AG, Clinton S, Stephens JK. A case report of three synchronous stage I malignant neoplasms. Gynecol Oncol. 1989 Apr;33(1):116-20. doi: 10.1016/0090-8258(89)90615-x
62. Eren MS, Ozdogan O, Koyuncuoglu M, Degirmenci B. Three synchronous primary tumors with different histology detected by ^{18}F-FDG PET/CT. Hell J Nucl Med. 2012 Jan-Apr;15(1):56-8
63. Patel S, Alfonso AE, Landis J, Suarez J. Three synchronous multiorgan primary cancers. All stage I. Arch Surg. 1985 Oct;120(10):1182-4. doi: 10.1001/archsurg.1985.01390340078016
64. Katz H, Jafri H, Brown L, Pacioles T. Triple synchronous primary malignancies: a rare occurrence. BMJ Case Rep. 2017 Jun 5;2017:bcr2017219237. doi: 10.1136/bcr-2017-219237

65. Li ZK, Zhao Q, Li NF, Wen J, Tan BX, Ma DY, Du GB. Synchronous triple primary malignant tumours in the bladder, prostate, and lung harbouring TP53 and MEK1 mutations accompanied with severe cardiovascular diseases: A case report. Open Med (Wars). 2022 Dec 14;17(1):2046-2051. doi: 10.1515/med-2022-0616
66. Kurul S, Akgun Z, Saglam EK, Basaran M, Yucel S, Tuzlali S. Successful treatment of triple primary tumor. Int J Surg Case Rep. 2013;4(11):1013-6. doi: 10.1016/j.ijscr.2013.08.010. Epub 2013 Aug 27
67. Kataoka S, Omae M, Horiuchi Y, Ishiyama A, Yoshio T, Hirasawa T, Yamamoto Y, Tsuchida T, Fujisaki J, Yamada K, Igarashi M. Synchronous triple primary cancers of the pharynx and esophagus. Clin J Gastroenterol. 2017 Jun;10(3):208-213. doi: 10.1007/s12328-017-0734-3
68. Peng L, Zeng Z, Teng X, Chen Z, Lin L, Bao H, Shao YW, Wang Y, Dong Y, Zhao Q. Genomic profiling of synchronous triple primary tumors of the lung, thyroid and kidney in a young female patient: A case report. Oncol Lett. 2018 Nov;16(5):6089-6094. doi: 10.3892/ol.2018.9334
69. Oey O, Tiong SS, Wong SL, Navadgi S, Khan Y. Triple synchronous malignancies of the stomach, bladder and thyroid in a previously treated prostate cancer patient: A Case Report. Folia Med (Plovdiv). 2023 Aug 31;65(4):693-698. doi: 10.3897/folmed.65.e96012
70. Oh SJ, Bae DS, Suh BJ. Synchronous triple primary cancers occurring in the stomach, kidney, and thyroid. Ann Surg Treat Res. 2015 Jun;88(6):345-8. doi: 10.4174/astr.2015.88.6.345
71. Qiu B, Lin C, Wu L, Li Y. A case report of synchronous triple primary malignancies: Diffuse large B-cell lymphoma, rectal adenocarcinoma and hepatocellular carcinoma. Front Oncol. 2022 Dec 21;12:1046878. doi: 10.3389/fonc.2022.1046878
72. Song X, Shen H, Li J, Wang F. Minimally invasive resection of synchronous triple primary tumors of the esophagus, lung, and thymus: A case report. Int J Surg Case Rep. 2016;29:59-62. doi: 10.1016/j.ijscr.2016.10.048
73. Lee JS, Moon W, Park SJ, Park MI, Kim KJ, Jang LL, Park MJ, Chun BK. Triple synchronous primary cancers of rectum, thyroid, and uterine cervix detected during the workup for hematochezia. Intern Med. 2010;49(16):1745-7. doi: 10.2169/internalmedicine.49.3549
74. Mendez LE, Atlass J. Triple synchronous primary malignancies of the colon, endometrium and kidney in a patient with Lynch syndrome treated via minimally invasive techniques. Gynecol Oncol Rep. 2016 May 25;17:29-32. doi: 10.1016/j.gore.2016.05.007
75. Huang R, Li Z, Weng S, Wu S. Simultaneous triple primary malignancies, including bladder cancer, lymphoma, and lung cancer, in an elderly male: A case report. Open Life Sci. 2022 Sep 26;17(1):1263-1268. doi: 10.1515/biol-2022-0500
76. Park CL, Moria F, Saleh RR. Combination of Osimertinib with Concurrent Chemotherapy and Hormonal Therapy for Synchronous NSCLC, Hormone Receptor-Positive Breast Cancer, and Triple-Negative Breast Cancer: Case Report. Case Rep Oncol. 2023 Oct 11;16(1):1080-1086. doi: 10.1159/000533783
77. Ágoston EI, Somorácz Á, Madaras L, Zaránd A, Szentmártoni G, Orosz Z, Dank M, Baranyai Z. Successful treatment of three synchronous primary malignant tumours-reflection on surgical, pathological and oncological aspects and decision making. J Surg Case Rep. 2018 Apr 6;2018(4):rjy041. doi: 10.1093/jscr/rjy041
78. Rai RS, Deb P, Rai R, Gupta E, Panayach JS. Synchronous primary triple neoplasia (renal cell carcinoma and prostate cancer in combination with thyroid neoplasm). Report of an unusual case. Minerva Urol Nefrol. 2007 Dec;59(4):451-4
79. Lee E, Ji YI. A Case of Uterine Carcinosarcoma Detected Simultaneously with Breast and Colon Cancer (Triple Primary Malignant Tumor). Case Rep Oncol. 2018 Jun 29;11(2):431-435. doi: 10.1159/000489844
80. AlBaqmi KH, AlMudaiheem FA, Boghdadly S, AlHussaini KA, Shokor N, AlOudah N. Multiple Primary Malignancies of the Colon, Stomach, and Kidney in a Patient with Bowel Obstruction Requiring Emergency Surgery: A Case Report. Am J Case Rep. 2020 Nov 27;21:e926472. doi:

10.12659/AJCR.926472
81. Jin CJ, Mei X, Falkson CB. A case of synchronous breast and bilateral lung cancers: literature review and considerations for radiation treatment planning. BJR Case Rep. 2015 Aug 25;3(1):20150464. doi: 10.1259/bjrcr.20150464
82. Jeon SY, Ahn SH, Kim CH, Lim SM, Koh JS, Lee JC. Esophageal and laryngeal cancer incidentally found on [18F]fluorodeoxyglucose positron emission tomography/computed tomography during the staging workup for lung cancer. Clin Lung Cancer. 2008 Jul;9(4):230-1. doi: 10.3816/CLC.2008.n.035
